# Adaptability and clinical applicability of UFS-QoL in Chinese women with uterine fibroid

**DOI:** 10.1186/s12905-022-01963-z

**Published:** 2022-09-10

**Authors:** Wei Xu, Wenzhi Chen, Jinyun Chen, Liang Hu, Xueyao Su, Yuxian Nie, Qiuling Shi

**Affiliations:** 1 School of Public Health, Chongqing Medical University, No. 1 Yixueyuan Road, Yuzhong District, Chongqing, 400016 People’s Republic of China; 2 State Key Laboratory of Ultrasound in Medicine and Engineering, Chongqing Medical University, Chongqing, People’s Republic of China; 3 Chongqing Haifu Hospital, Chongqing, People’s Republic of China; 4 College of Biomedical Engineering, Chongqing Medical University, Chongqing, People’s Republic of China

**Keywords:** Clinical applicability, Health-related quality of life, Uterine fibroids, UFS-QoL

## Abstract

**Objective:**

To demonstrate the applicability and adaptability of uterine fibroid symptoms and quality of life (UFS-QoL) in assessing the efficacy of treatment in Chinese populations.

**Methods:**

This is a secondary analysis of a prospective cohort study involving 20 Chinese hospitals and 2,411 Chinese women with fibroids. Patients completed UFS-QoL and short form-36 (SF-36) at pre-surgery, 6-month and 12-month post-treatments. Internal consistency of the quality of life assessed by the UFS-QoL questionnaire using Cronbach’s α coefficient (α). Principal axis factor analysis with orthogonal rotation was established to investigate relationships between items and subscales. Concurrent validity refers to the Spearman's correlation estimate of the correlation between UFS-QoL and SF-36. Using effect size and standardized response mean, the ability to detect change was evaluated by comparing pre- and post-6-month and post-12-month treatment scores.

**Results:**

Exploratory factor analysis yielded six subscales (concern, activities, energy/mood, control, self-consciousness, and sexual function) with eigenvalues > 1 in UFS-QoL. A 63.61% total variance was explained by the test items. Ceiling effects of self-consciousness and sexual functioning subscales from UFS-QoL were > 15%. UFS-QoL showed a positive and moderate correlation with SF-36 to establish good concurrent validity. And showed good consistency reliability (Cronbach α > 0.7 in all subscales), ability to detect change after treatment. This excluded self-consciousness (α = 0.56), which demonstrated the lowest effect size (0.38) and standardized response means (0.38) 6- and 12-months post-treatment.

**Conclusions:**

Symptom severity, activity, and mood subscales of the Chinese UFS-QoL were valid and reliable. However, the self-consciousness domain needs further investigation on cultural adaptation, such as cognitive debriefing for how Chinese interpret these questions.

**Supplementary Information:**

The online version contains supplementary material available at 10.1186/s12905-022-01963-z.

## Highlights

1. This study provides clinicians and researchers with more specific psychological evidence for selecting an appropriate instrument and demonstrate the benefit of accurately assessing the quality of life and symptom burden in Chinese patients with uterine fibroids.
2. In the Chinese UFS-QoL, the symptom severity, activity, and mood subscales were valid and reliable.
3. At 6 and 12 months after treatment, the self-consciousness domain of the UFS-QoL exhibited both poor internal consistency and detectability, requiring further research into cultural adaptation.

**Supplementary Information:**

The online version contains supplementary material available at 10.1186/s12905-022-01963-z.

## Introduction

Patient-centeredness has been increasingly recognized as a crucial aspect of patient care [1]. With an increased emphasis on patient-centered care, health-related quality of life (HRQL) that permits patient self-report is assuming a more prominent role as an important endpoint and a major treatment indicator in both research and clinical practice [2]. For an average of 8.5 years [3], patients with uterine fibroids (UFs) experience a high symptom burden, including moderate to severe abdominal pain [4], low back pain, urinary frequency and urgency, pain during intercourse [4], and vaginal bleeding [5], all of which negatively impact physical and social activities, work productivity, and quality of life (QoL) [6, 7]. Importantly, the lifetime risk of UFs for women over 45 is up to 60% [8]. Given the high prevalence among women, UFs impose a significant health care burden on women's individual health [9] as well as a burden on the health care and social security systems as a result of work productivity loss during treatment and disease recurrence [10].

As one of the direct clinical outcome assessments [11], patient-reported outcome (PRO)-based symptom and QoL measures serve as reliable approaches for patients with UFs throughout the full course of diagnosis [12], treatment [13], and disease management [14]. Consequently, measuring UF-related symptoms and QoL status in a valid and reliable manner could support high-quality practice and comprehensive patient management for patients of diverse cultures.

The uterine fibroid symptom and quality of life (UFS-QoL) English questionnaire was published in 2002 as the only questionnaire designed to assess the whole spectrum of fibroid-related symptoms and their impact on QoL [15]. The UFS-QoL questionnaire has been used in Brazilian, Portuguese [16, 17], Spanish [18], traditional Chinese [19], and simplified Chinese [20] as a disease-specific measure of health-related QoL. It has been shown to be sensitive to treatment-related changes [18], with the 4-week recall version being sensitive to treatment-related changes in Western culture [21]. Self-consciousness has the lowest Cronbach α based on existing UFS-QoL validation studies in Chinese populations, [20] possibly due to existing adaptive barriers in China. QoL is a subjective and multidimensional concept based on the individual's perception of the position of their life in the cultural context and value systems in which they reside, in relation to their goals, expectations, standards, and concerns [16]. In the evaluation of QoL, patient-related concepts that vary from person to person are considered [22]. However, social, linguistic, and cultural differences necessitate proper cultural adaptation.

The two Chinese validation studies only demonstrated that the UFS-QoL questionnaire can identify disease and symptoms in patients by comparing women with UFs and healthy women in a cross-sectional analysis [20, 23]. However, a few studies reported that PROs play an important role as clinical tools for clinical application and disease monitoring, such as differentiating disease severity and demonstrating symptom recovery and alleviation trajectory over time, not only screening for UFs. The responsiveness of the UFS-QoL, which is one of the major characteristics of Food and Drug Administration (FDA)-reviewed PRO instruments, must be further validated to determine its efficacy. Therefore, large longitudinal population-based studies are required to further evaluate the clinical utility of UFS-QoL.

We can demonstrate the measurement properties of UFS-QoL when longitudinally applied in clinical settings due to the availability of longitudinal UFS-QoL data from the largest cohort of patients with UFs ever. Using internal consistency, convergent validity, known-group validity, and concurrent validity (correlation between UFS-QoL and SF-36), the current study aimed to demonstrate the applicability and adaptability of the UFS-QoL in evaluating adaptability and treatment efficacy. We also evaluated responsiveness, the ability to detect change with treatment using UFS-QoL, such as when comparing the effect size of major UFs treatment modalities before and after treatment.

## Methods

Data of uterine fibroids were extracted from a 20-centered prospective cohort study (Uterine fibroids multicentre network information system: www.hifuctr.com) for a second-analysis, which included patients who received self-selected hysterectomy, myomectomy, or High-Intensity Focused Ultrasound (HIFU) therapy after being fully informed of the treatment options (The multicentre study was approved by a China-registered clinical trial ethics committee (ChiECRCT-2011034). Details regarding the study's design, data collection, and primary outcomes regarding the efficacy of the treatment were published [24]. Prior to undergoing any study-related procedures at the clinical site, patients filled out the UFS-QoL questionnaire, the study short form-36 (SF-36), and a brief sociodemographic questionnaire. Follow-up visits were scheduled at 6 months and 12 months post-procedure, included complications, magnetic resonance imaging evaluation, overall treatment effect evaluates, the UFS-QoL questionnaire (for those who had under-gone HIFU or myomectomy, because the instructions of the UFS-QoL questionnaire are based on the presence of uterine fibroids and menstrual periods), SF-36, and several health care utilization items were recorded.

### Questionnaires utilized in the study

Uterine fibroid symptom and quality of life questionnaire (UFS-QoL).

The UFS-QoL was developed from focus groups of women with uterine fibroid [15, 20]. The UFS-QoL questionnaire consists of 37 items, 8 of which assess the severity of symptoms (single domain) and 29 of which assess health-related quality of life (HRQL) in 6 subscales (concern, activities, energy/mood, control, self-consciousness, and sexual function). All responses were classified into five Likert scale options. A higher score on the questionnaire's severity subscale indicates more severe symptoms, while a lower score on the HRQL subscales indicates poorer QoL.

Medical outcomes of the study short form 36 (SF-36).

SF-36 is a 36-item self-administered generic measure used to assess general health status. and validated cross-cultural application, reality and validity in Chinese [25–28]. SF-36 consists of eight subscales: physical, functioning, role-physical, bodily pain, general health, vitality, social functioning, role-emotional, and mental health, as well as two composite scores, the physical and mental component scores. Individual subscale items are combined to form a subscale rating, which is then converted to a 0–100 scale [29, 30]. Higher QoL scores correspond to a four-week recall period [30]. There are available reference values derived from a healthy population and distributed by age and gender. We estimate of the Spearman's correlation between UFS-QoL and SF-36 to validate the concurrent validity.

### Statistical analysis

Descriptive analyses (mean and SD) were performed using sociodemographic and clinical characteristics. Means of differences, 95% confidence interval (CI), and statistical significance (P < 0.05) were tested with independent sample t tests.

Cronbach's α coefficient was applied to determine the internal consistency of the quality of life. The Cronbach's α coefficient, which ranges from 0 to 1, is used to determine the degree to which items on the 6-subscale HRQL measure are related to the same concepts. A greater value indicates a smaller measurement error, which indicates a higher level of reliability.

To examine convergent validity between items and subscales in our study, we employed principal axis factor analysis with orthogonal rotation, which was used in conjunction with orthogonal rotation to determine the final number of factors based on their eigenvalues, congruence, and clinical significance. The Kaiser–Meyer–Olkin (KMO) test confirmed adequate sample size, and a KMO value > 0.5 indicated an acceptable structural validity. With structural equation modeling (SEM) and orthogonal (intercorrelated) factors, a confirmatory factor analysis was conducted. Examining the relative chi-square (chi-square/degrees of freedom), the root mean-square error of approximation, the goodness of fit index, the standardized root means square residual, the normed fit index, the Tucker Lewis index, and the comparative fit index allowed for the evaluation of model fit. The expected factor loadings for each item were ≥ 0.40 [31].

Concurrent validity refers to the correlation between an instrument and another instrument that measures a related but not identical concept, and it is used to evaluate the reliability of correlation testing of a priori hypotheses [32]. Both scales were designed to measure QoL, so Spearman's correlation was used to determine the strength of the UFS-correlation QoL's testing with SF-36 to validate concurrent validity. Similar UFS-QoL and SF-36 subscales were correlated. For instance, the SF-36's "physical functioning domain" was compared to the UFS-QoL's “activity.” The SF-36 "role-emotional" domain was compared with the UFS-QoL "mood/energy" domain.

We examined evidence of known-group validity that the UFS-QoL can distinguish between clinically distinct groups by testing its ability to differentiate between patients based on health status. Patients were considered to have a poor general health status if they responded "fair" or "poor" to the SF-36–1 question “In general, would you say your health is”; otherwise, the health status was presumed good.

Using general linear models, the ability to detect change was evaluated by comparing 6-month pre-treatment and post-treatment scores to 12-month scores at 6-month intervals. Effect size (change in mean score divided by baseline standard deviation) [33] and standardized response mean (change in mean score divided by change standard deviation) were computed. A value of 0.2 was considered to have a “small” effect, 0.5 a “moderate” one, and ≥ 0.8 a “large” one.

The questionnaires were scored in accordance with the developers' instructions. Version 9.1.3 of SAS was used to conduct analyses. All statistical tests were predetermined, and no missing data imputations were performed. All statistical tests were conducted with a fixed type I error probability of 0.05 and a two-tailed design [29].

## Results

### Psychometric characteristics

To assess internal consistency, Cronbach's alphas were calculated for each UFS-QoL questionnaire subscale. The UFS-QoL questionnaire demonstrated good internal consistency and reliability in all subscales (> 0.7) at baseline and follow-up (6 and 12 months) except for self-consciousness (0.5–0.62) (Table 1). We also calculated the internal consistency of self-consciousness stratified by age (< 45 vs. ≥ 45), highest educational level (below junior school vs. above junior school), annual family income (< 50,000 vs. ≥ 50,000), and number of pregnancies (1 vs. > 1), which all depicted a Cronbach’s α < 0.7 (Additional file 1: Table S1).

**Table 1:** Internal consistency reliability of UFS-QOL subscales

UFS-QOL	k	Internal consistency (Cronbach’s alpha)*									
		Baseline				6-month			12-month		
		All	HIFU	Myomectomy	Hysterectomy	All	HIFU	Myomectomy	All	HIFU	Myomectomy
Symptom severity	8	0.78	0.79	0.78	0.77	0.80	0.8	0.75	0.83	0.83	0.79
Concern	5	0.82	0.81	0.84	0.85	0.81	0.81	0.82	0.84	0.83	0.84
Activities	7	0.85	0.86	0.82	0.86	0.84	0.84	0.83	0.84	0.85	0.81
Energy/mood	7	0.89	0.89	0.88	0.89	0.87	0.88	0.87	0.87	0.88	0.85
Control	5	0.77	0.79	0.73	0.76	0.76	0.76	0.78	0.77	0.77	0.76
Self-consciousness	3	**0.56**	**0.57**	**0.57**	**0.55**	**0.59**	**0.60**	**0.56**	**0.59**	**0.59**	**0.58**
Sexual functioning	2	0.87	0.86	0.88	0.86	0.88	0.86	0.91	0.86	0.84	0.9
Total HRQL score	29	0.95	0.95	0.93	0.95	0.95	0.95	0.94	0.95	0.95	0.94

k, number of items

*UFS-QOL* Uterine Fibroid Symptom and Health-related Quality of Life, *HRQL* Health-related Quality of Life, *HIFU* high-intensity focused ultrasound

*> 0.70 = satisfactory internal consistency

The factor analysis was utilized to determine the relative importance of UFS-QoL components. In factor analysis, values of the KMO test > 0.7 and the statistical significance of the Bartlett's test indicate adequate sampling [34, 35]. We used orthogonal rotation to isolate the potential UFS-QoL factors (Table 2). In this study, the value of KMO was 0.954, and the value of Bartlett's sphericity test was 2,536.26 (P < 0.001), indicating that factor analysis was suitable for the data. The UFS-QoL retained six factors. Overall, the test variables explained 63.61% of the total variance. 11.68, 1.75, 1.61, 1.20, 1.13, and 1.07 were the eigenvalues of the factors, and the variance percentages of the test were 40.27, 6.04, 5.54, 4.15, 3.91, and 3.07. Five items have a factor load of greater than 0.40 on multiple factors, and their item numbers are 12, 14, 16, 19, 29; meanwhile, two items load on any factor below or equal to 0.40. The item numbers for these items are 26 and 27. Additional file 1: Table S2 displays the distribution of items within each factor, the comparison with the subscales of the original questionnaire, and the Chinese version of the original validation. Comparative fit index of 0.842, Tucker Lewis index of 0.894, and root mean square error of approximation of 0.077 for this six-factor confirmatory factor analysis model indicate adequate model fit (Additional file 1: Table S3).

**Table 2:** Construct validity of the HRQL: baseline factor loadings of the HRQL items (N = 2411)

HRQL item	Factor1	Factor2	Factor3	Factor4	Factor5	Factor6
U_24	**0.78362**	0.19066	0.20176	0.04472	0.15800	0.06729
U_23	**0.74291**	0.21319	0.19319	0.00739	0.20221	0.10002
U_25	**0.70961**	0.28565	0.19429	0.11984	0.05806	0.09629
U_31	**0.66866**	0.02244	0.27166	0.19889	0.07939	0.11970
U_35	**0.61022**	0.27571	0.09054	0.25979	0.31465	0.11369
U_30	**0.57827**	0.13125	0.15194	0.17442	0.38899	0.06520
U_34*	**0.56625**	0.19640	0.07072	0.24148	0.47445	0.09197
U_17	**0.53628**	0.31827	0.21665	0.19449	-0.22987	0.30472
U_19*	**0.52409**	0.42514	0.15739	0.15920	-0.02111	0.35012
U_26^†^	**0.38574**	0.07028	0.37443	0.31548	-0.08992	0.04264
U_27^†^	**0.35316**	0.27929	0.34406	0.16690	0.23795	0.17964
U_11	0.14414	**0.79409**	0.14252	0.09529	0.16389	0.08916
U_13	0.25134	**0.71928**	0.12842	0.11221	0.00710	0.17708
U_10	0.07971	**0.70103**	0.19178	0.02326	0.34197	0.02452
U_9	0.13350	**0.60268**	0.31085	0.10547	0.06850	-0.06081
U_12*	0.52405	**0.54563**	0.17555	0.20402	-0.08179	0.12979
U_20	0.37788	**0.53253**	0.06939	0.11783	0.23488	0.28056
U_14*	0.44900	**0.52848**	0.02547	0.05679	0.30930	0.05665
U_16*	0.46475	**0.48823**	0.20467	0.14324	-0.01209	0.18487
U_22	0.19534	0.14872	**0.80968**	0.07997	0.04101	0.19420
U_15	0.18225	0.27956	**0.78000**	0.06666	0.01635	0.06019
U_32	0.20872	0.08866	**0.77244**	0.09216	0.21949	0.04500
U_28	0.19559	0.27739	**0.55080**	0.15950	0.39276	0.07063
U_29*	0.21884	0.24856	**0.50269**	0.09154	0.48746	0.12506
U_36	0.2415	0.13704	0.14093	**0.85710**	0.09273	0.11595
U_37	0.19862	0.18721	0.14051	**0.84206**	0.16299	0.10297
U_33	0.18518	0.22976	0.2539	0.09808	**0.59398**	0.21672
U_18	0.13972	0.05445	0.12288	0.07548	0.04715	**0.79176**
U_21	0.15293	0.16183	0.09861	0.09261	0.19291	**0.72138**

The bold type means HRQL items are assigned to the corresponding factor through factor analysis

Kaiser–Meyer–Olkin (KMO): 0.954

Total variance explained: 63.6%

Values in bold indicate that they belong to the same factor

*UFS-QOL* Uterine Fibroid Symptom and Health-related Quality of Life, *HRQL* Health-related Quality of Life

*The item load on any factor greater than 0.40 on more than one factor: U_19, U_12, U_14, U_16, U_29

^†^The item load on any factor less than or equal to 0.40: U_26, U_27

As shown in Additional file 1: Table S4, floor or ceiling effects are present if > 15% of respondents achieved the lowest or highest possible score, respectively [36]. In six subscales, ceiling effects ranged from 5.14% to 16.96%, while self-consciousness (15.18%) and sexual functioning (16.96%) were all > 15%. The ceiling effect varies between 0.02 and 0.5%.

Assessing the degree of correlation between similar subscales on the UFS-QoL and SF-36 was used to assess the reliability of correlation testing for the UFS-QoL (Table 3). The “physical functioning” domain of the SF-36 correlated positively and moderately with the “activity” domain of the UFS-QoL (r: 0.33–0.4, P < 0.001). The “role-emotional” domain of the SF-36 had a moderately positive correlation with the “energy/mood” domain (r: 0.35–0.43, P < 0.001). Similarly, the “role-physical” subscale of SF-36 had a moderate correlation with the “control” subscale of the UFS-QoL (r: 0.3–0.4, P < 0.001).

**Table 3:** Relationship of UFS-QoL Subscale Scores and SF-36*

	PF	PR	BP	GH	VT	SF	ER	MH
*Baseline*								
Symptom severity	− 0.33	− 0.32	− 0.32	− 0.28	− 0.31	− 0.26	− 0.29	− 0.25
Concern	0.24	0.26	0.23	0.30	0.30	0.30	0.26	0.26
Activities	0.40	0.43	0.39	0.42	0.44	0.51	0.41	0.40
Energy/mood	0.36	0.41	0.36	0.47	0.58	0.48	0.43	0.53
Control	0.34	0.40	0.36	0.47	0.5	0.48	0.43	0.47
Self-consciousness	0.26	0.26	0.27	0.29	0.35	0.34	0.29	0.35
Sexual functioning	0.31	0.3	0.24	0.33	0.34	0.32	0.30	0.33
Total HRQL score	0.39	0.43	0.38	0.47	0.52	0.5	0.44	0.47
*Treatment Month 6*								
Symptom severity	− 0.27	− 0.24	− 0.32	− 0.27	− 0.29	− 0.19	− 0.31	− 0.21
Concern	0.24	0.19	0.25	0.26	0.28	0.21	0.20	0.27
Activities	0.37	0.35	0.36	0.37	0.39	0.37	0.33	0.36
Energy/mood	0.32	0.32	0.35	0.41	0.46	0.31	0.35	0.41
Control	0.32	0.32	0.34	0.45	0.43	0.32	0.33	0.37
Self-consciousness	0.28	0.23	0.28	0.26	0.28	0.20	0.24	0.27
Sexual functioning	0.24	0.19	0.25	0.31	0.32	0.20	0.23	0.25
Total HRQL score	0.36	0.33	0.37	0.42	0.44	0.33	0.34	0.39
*Treatment Month 12*								
Symptom severity	− 0.37	− 0.28	− 0.38	− 0.36	− 0.32	− 0.23	− 0.32	− 0.27
Concern	0.32	0.21	0.21	0.30	0.25	0.20	0.26	0.24
Activities	0.39	0.32	0.33	0.39	0.40	0.34	0.36	0.36
Energy/mood	0.38	0.33	0.32	0.42	0.44	0.29	0.39	0.40
Control	0.37	0.30	0.31	0.44	0.38	0.29	0.36	0.35
Self-consciousness	0.29	0.24	0.26	0.30	0.28	0.18	0.29	0.26
Sexual functioning	0.28	0.24	0.26	0.36	0.33	0.25	0.31	0.29
Total HRQL score	0.41	0.33	0.34	0.44	0.42	0.32	0.39	0.38

HRQL, Health-related Quality of Life; PF, Physical Functioning; RP, Role-physical; BP, Bodily Pain; GH, General Health; VT, Vitality; SF, Social Functioning; RE, Role-emotional; MH, Mental Health

*Spearman’s correlations: all P < 0.0001

UFS-QoL was sensitive enough to detect varying levels of current health status, particularly six and twelve months after surgery (Table 4). Patients with poor general health status (those who rated "In general, would you say your health" as fair or poor on the SF-36-1) had statistically significantly higher severity for all symptoms and poorer QoL than those with good general health status (those who rated "In general, would you say your health" as excellent, very good, or good) (all P < 0.0001), and all effect sizes > 0.5 indicated a moderate effect 6 and 12 months after surgery.

**Table 4:** Known-group validity: comparison of UFS-QOL symptom and quality of life scores and known health status

	Baseline				6-month				12-month			
	Good^a^	Poor^b^	Mean difference^c^	Effect size^d^	Good	Poor	Mean difference	Effect size	Good	Poor	Mean difference	Effect size
N	1564	847			1548	301			1557	215		
symptom severity	16.73 ± 13.06	22.16 ± 15.59	− 5.4 (− 6.6 to − 4.3)	− 0.35*	7.94 ± 8.25	16.11 ± 13.31	− 8.2 (− 9.3 to − 7)	− 0.62*	5.81 ± 7.34	16.65 ± 14.11	− 10.8 (− 12 to− 9.6)	− 0.77*
Concern	69.29 ± 20.99	62.89 ± 21.52	6.4 (4.6–8.2)	0.30*	80.6 ± 15.51	70.23 ± 19.37	10.4 (8.4–12.4)	0.54*	84.55 ± 14.5	72.44 ± 20.82	12.1 (9.9–14.3)	0.58*
Activities	78.03 ± 17.35	69.35 ± 17.98	8.7 (7.2–10.2)	0.48*	85.63 ± 12.48	75.14 ± 16.45	10.5 (8.9–12.1)	0.64*	88.54 ± 11.18	75.91 ± 17.83	12.6 (10.9–14.4)	0.71*
Energy/mood	76.12 ± 16.74	65.44 ± 19.48	10.7 (9.2–12.2)	0.55*	83.67 ± 13.09	69.46 ± 18.29	14.2 (12.5–15.9)	0.78*	86.56 ± 11.62	70.37 ± 18.92	16.2 (14.4–18)	0.86*
Control	75.88 ± 15.64	66.56 ± 18.15	9.3 (7.9–10.7)	0.51*	83.89 ± 12.09	70.83 ± 17.89	13.1 (11.4–14.7)	0.73*	86.67 ± 11.19	71.49 ± 18.62	15.2 (13.4–16.9)	0.82*
Self-consciousness	78.07 ± 17.59	71.15 ± 19.92	6.9 (5.4–8.5)	0.35*	84.36 ± 14.88	74.81 ± 17.59	9.6 (7.7–11.5)	0.55*	86.58 ± 13.21	73.41 ± 18.64	13.2 (11.2–15.2)	0.71*
Sexual functioning	72.07 ± 21.59	63.72 ± 21.88	8.3 (6.5–10.2)	0.38*	82.63 ± 18.93	67.77 ± 24.99	14.9 (12.4–17.3)	0.60*	86.52 ± 16.59	67.73 ± 25.38	18.8 (16.2–21.3)	0.74*
Total HRQL score	75.52 ± 14.67	66.99 ± 16.1	8.5 (7.3–9.8)	0.53*	83.81 ± 11.32	71.97 ± 15.3	11.8 (10.4–13.3)	0.77*	86.81 ± 10.17	72.62 ± 16.1	14.2 (12.6–15.8)	0.88*

The bold type means HRQL items are assigned to the corresponding factor through factor analysis

*HRQL* Health-related Quality of Life

^a^Good health status: patients reported SF-36–1 “In general, would you say your health is excellent, very good, and good”

^b^Poor health status: patients reported fair, or poor

^c^mean difference: Good health status vs. Poor health status

^d^A value of 0.2 was considered as a ‘small’ effect, 0.5 a ‘moderate’ effect, and ≥ 0.8 a ‘large’ effect

^*^P < 0.001

Except for self-consciousness 6 and 12 months after surgery, UFS-QoL items displayed a good ability to detect change in general (Table 5). After treatment, there was a significant decrease in symptom severity scores and an improvement in HRQL subscale scores. Mean score change from baseline to 12-month follow-up for symptom severity was − 11.5 (P < 0.001), with an effect size of − 0.81 and standardized response means (SRM) of − 0.81 (Table 5). The mean change in score for the HRQL subscales ranged from 7.20 (sexual function) to 11.70 (concern), with effect sizes ranging from 0.38 (self-consciousness) to 0.55 (concern) and SRM ranging from 0.38 (self-consciousness) to 0.6 (concern) in 6 months after treatment. The self-consciousness subscale exhibited the lowest effect size (0.38), as well as the lowest SRM (0.38).

**Table 5:** Responsiveness of women with uterine fibroids after treatment

Subscales	Baseline	Mean score (SD)		Change in score (SD)		Effect size*		SRM^†^	
		6-month	12-month	6-month	12-month	6-month	12-month	6-months	12-months
Symptom severity	18.64 (14.24)	9.27 (9.74)	7.12 (9.15)	− 9.42 (13.29)	− 11.5 (14.21)	− 0.66	− 0.81	− 0.71	− 0.81
Concern	67.04 (21.4)	78.9 (16.63)	83.09 (15.89)	11.7 (19.35)	15.79 (22.06)	0.55	0.74	0.6	0.72
Activities	74.98 (18.05)	83.92 (13.75)	87.01 (12.85)	8.53 (16.44)	11.55 (18.53)	0.47	0.64	0.52	0.62
Energy/mood	72.38 (18.47)	81.34 (15)	84.61 (13.77)	9.04 (17.01)	12.4 (19.17)	0.49	0.67	0.53	0.65
Control	72.61 (17.15)	81.76 (14.05)	84.84 (13.28)	8.9 (16.37)	11.94 (18.59)	0.52	0.7	0.54	0.64
Self-consciousness	75.65 (18.73)	82.8 (15.74)	84.99 (14.62)	7.2 (19.14)	8.84 (20.71)	0.38	0.47	0.38	0.43
Sexual functioning	69.14 (22.05)	80.19 (20.77)	84.24 (18.9)	9.3 (23.2)	13.17 (25.73)	0.42	0.6	0.4	0.51
Total HRQL score	72.52 (15.73)	81.88 (12.81)	85.1 (11.99)	9.18 (13.75)	12.38 (16.04)	0.58	0.79	0.67	0.77

*HRQL* health-related quality of life, *SD* standard deviation, *SRM* standardized response mean

*Calculated as change in mean score divided by the standard deviation of the baseline

^†^Calculated as change in mean score divided by the standard deviation of the change

## Discussion

This is the first study from the largest cohort of patients with UFs to evaluate the adaptability and clinical applicability of the UFS-QoL in comparative effectiveness research involving clinical manifestations with varying severity. It further evaluated the UFS-QoL's capacity to produce valid and consistent results. Our study revealed that the Chinese version of the UFS-QoL requires further modification for cross-cultural adaptation, and that ongoing efforts in the management of uterine fibroids in Chinese must be expanded to reduce disparities in individual symptom burdens. This study will aid clinicians and researchers in selecting a suitable instrument for measuring quality of life and symptom burden in Chinese patients with uterine fibroids.

In this analysis, we found that some items did not adequately affect any factor (≤ 0.40 or ≥ 0.40 on more than one factor) in the factor analysis, which, according to the decision rules for item reduction in development studies [15], resulted in differences in the factor subscales from the original subscales. These differences may be attributable to cultural context and language system differences between China and other countries. This study and others have demonstrated that "Caused you embarrassment?" belongs to the concern subscales, not the activities subscales. Meanwhile, "Made you feel anxious about the unpredictable onset or duration of your periods?" belongs to activities unrelated to menstruation. Some Chinese questions containing both energy and activity subscales, such as "Item 19: Made you feel it was difficult to carry out your usual activities," resulted in inadequate item discrimination. China with a complex population structure and a large gap between the rich and the poor, both of which have varied effects on cross-cultural tests [16]. Lacking are cognitive debriefing or linguistic validation in Chinese population validation [19, 20, 23], and cross-cultural tests to eliminate cultural differences. Before applying the UFS-QoL to Chinese populations, the cross-cultural test criteria of adjusting the subscales to the Chinese culture, shortening or deleting the poor discrimination items to ensure the validity of the scale, and further optimizing and reevaluating the practicability of these items, should be considered.

FDA claims that a PRO questionnaire as an instrument should measure what it is intended to measure, and it is assumed that cross-cultural adaptation will yield an equivalent measure [37]. Self-consciousness possessed the lowest Cronbach's α, ceiling effects, and responsiveness. This subscale contains three questions: (1) Made you feel self-conscious of weight gain; (2) Made you feel conscious about the size and appearance of your stomach? (3) Affected the size of clothing you wear during your periods. Our findings indicate that these three questions may not be sensitive enough to assess the concept of interest and self-consciousness in Chinese patients with UFs, despite their content validity in western populations [15, 17]. When applying UFS-QoL to Chinese populations, it is necessary to modify the item definition and concept of reliability and sensitivity by incorporating cognitive debriefing, focus groups and/or committees, Rasch measurement theory analysis, and traditional psychometrics.

Sexual health and health-related QoL may be phenomenologically related [38]. However, sexual functioning has demonstrated poor responsiveness and a significant ceiling effect, which may be attributable to differences in study design and culture. Sexuality is openly discussed on the mainland. China remains controversial, and research in this area is relatively new [39], which casts doubt on the authenticity of certain objects. To determine how uterine fibroids affect sexual activity in Chinese populations, it is necessary to adapt most of the sex QoL questionnaires validated in China, from frequency and level subscales to the evaluation of sexual functioning [40].

Our study's strengths include a sufficient sample size and the incorporation of multiple treatments, thereby validating the Chinese version of the UFS-QoL for myomectomy, HIFU, and hysterectomy. Another strength of our study is that we found that the self-consciousness domain requires additional research into cultural adjustment, as it had the lowest Cronbach's α (0.56), effect size (0.38), and SRM (0.38). This study had a few limitations. First, we evaluated responsiveness and comparative efficacy using effect size instead of a P value, yielding some small effect results. Second, there was no follow-up for the UFS-QoL questionnaire in the hysterectomy group, as the questionnaire's instructions are contingent on the presence of menstrual periods. Thirdly, we did not include healthy women in the analysis because the purpose of the clinical application was not to diagnose and differentiate uterine fibrous patients from women without myoma; rather, we focused on the clinical evaluation, including the severity of the illness.

## Conclusion

In the Chinese version of the UFS-QoL, the symptom, activity, and mood interference subscales were culturally appropriate and reliable. However, the self-consciousness domain requires additional research on cultural adaptation, such as cognitive debriefing of how Chinese populations interpret the questions. This study will provide clinicians and researchers with more specific psychological evidence for selecting an appropriate instrument and demonstrate the benefit of accurately assessing the quality of life and symptom burden in Chinese patients with uterine fibroids. The results of the self-consciousness subscale of the UFS-QoL should be interpreted with caution when evaluating the quality of life of patients with uterine fibroids.

## Supplementary Information

**Additional file 1. Table S1**. Internal consistency of Self-consciousness stratifies by demographics (Cronbach’s alpha)*.

## Data Availability

All data in the main text and the Supplementary Information have been uploaded into a website (www.hifuctr.com). Readers have read access to the data but are not allowed to export the data. Full access to the data will be available to researchers for the purposes of research or regulatory decision-making with a signed data access agreement after approval of a proposal. All data requests will be reviewed by the research committee at Chongqing Medical University and the corresponding authors to verify whether the request is subject to any intellectual property or confidentiality obligations.

## Abbreviations

UFS-QoL: Uterine Fibroid Symptom and Quality of Life
HRQL: Health-Related Quality of Life
Ufs: Uterine Fibroids
PRO: Patient-reported outcome
FDA: Food and Drug Administration
SF-36: Short Form-36
HIFU: High-Intensity Focused Ultrasound
KMO: Kaiser–Meyer–Olkin
RMSEA: Root Mean Square Error of Approximation
TLI: Tucker Lewis Index
CFI: Comparative Fit Index
PF: Physical Functioning
RP: Role-physical
BP: Bodily Pain
GH: General Health
VT: Vitality
SF: Social Functioning
RE: Role-emotional
MH: Mental Health
SD: Standard Deviation
SRM: Standardized Response Means

## References

[1] Institute of Medicine Committee on Quality of Health Care (2001). Crossing the quality chasm: a new health system for the 21st century.

[2] Lemieux J, Goodwin PJ, Bordeleau LJ, Lauzier S, Théberge V (2011). Quality-of-life measurement in randomized clinical trials in breast cancer: an updated systematic review (2001–2009). J Natl Cancer Inst.

[3] Marsh EE, Al-Hendy A, Kappus D, Galitsky A, Stewart EA, Kerolous M (2018). Burden, prevalence, and treatment of uterine fibroids: a survey of U.S. women. J Women's Health (2002).

[4] Deal LS, Williams VS, Fehnel SE (2011). Development of an electronic daily uterine fibroid symptom diary. Patient.

[5] Egbe TO, Badjang TG, Tchounzou R, Egbe EN, Ngowe MN (2018). Uterine fibroids in pregnancy: prevalence, clinical presentation, associated factors and outcomes at the Limbe and Buea Regional Hospitals, Cameroon: a cross-sectional study. BMC Res Notes.

[6] Ghant MS, Sengoba KS, Recht H, Cameron KA, Lawson AK, Marsh EE (2015). Beyond the physical: a qualitative assessment of the burden of symptomatic uterine fibroids on women's emotional and psychosocial health. J Psychosom Res.

[7] Laberge PY, Vilos GA, Vilos AG, Janiszewski PM (2016). Burden of symptomatic uterine fibroids in Canadian women: a cohort study. Curr Med Res Opin.

[8] Stewart EA, Cookson CL, Gandolfo RA, Schulze-Rath R (2017). Epidemiology of uterine fibroids: a systematic review. BJOG Int J Obstet Gynaecol.

[9] Whiteman MK, Kuklina E, Jamieson DJ, Hillis SD, Marchbanks PA (2010). Inpatient hospitalization for gynecologic disorders in the United States. Am J Obstet Gynecol.

[10] Baird DD, Patchel SA, Saldana TM, Umbach DM, Cooper T, Wegienka G, Harmon QE (2020). Uterine fibroid incidence and growth in an ultrasound-based, prospective study of young African Americans. Am J Obstet Gynecol.

[11] Gabrilove JL, Perez EA, Tomita DK, Rossi G, Cleeland CS (2007). Assessing symptom burden using the M. D. Anderson symptom inventory in patients with chemotherapy-induced anemia: results of a multicenter, open-label study (SURPASS) of patients treated with darbepoetin-at a dose of 200 μg every 2 weeks. Cancer.

[12] Zhou XJ, Zhao ZM, Liu P, Zhao CY, Lin YJ, Liu Y, Wang M, Tian C, Li HY, Hou CX (2020). Efficacy of high intensity focused ultrasound treatment for cystic adenomyosis: a report of four cases. Ann Palliat Med.

[13] Ma J, Brown B, Liang E (2021). Long-term durability of uterine artery embolisation for treatment of symptomatic adenomyosis. Aust N Z J Obstet Gynaecol.

[14] Tinelli A, Gustapane S, D'Oria O, Licchelli M, Panese G (2021). Nutraceuticals in fibroid management after ulipristal acetate administration: an observational study on patients' compliance. Int J Gynaecol Obstet.

[15] Spies JB, Coyne K, Guaou Guaou N, Boyle D, Skyrnarz-Murphy K, Gonzalves SM (2002). The UFS-QOL, a new disease-specific symptom and health-related quality of life questionnaire for leiomyomata. Obstet Gynecol.

[16] Silva RO, Gomes MT, Castro RA, Bonduki CE, Girão MJ (2016). Uterine Fibroid Symptom—Quality of Life questionnaire translation and validation into Brazilian Portuguese. Revista brasileira de ginecologia e obstetricia: revista da Federacao Brasileira das Sociedades de Ginecologia e Obstetricia.

[17] Oliveira Brito LG, Malzone-Lott DA, Sandoval Fagundes MF, Magnani PS, Fernandes Arouca MA, Poli-Neto OB, Nogueira AA (2017). Translation and validation of the Uterine Fibroid Symptom and Quality of Life (UFS-QOL) questionnaire for the Brazilian Portuguese language. Sao Paulo Med J = Revista paulista de medicina.

[18] Calaf J, Palacios S, Cristóbal I, Cañete ML, Monleón J, Fernández J, Hernández A, Vázquez F (2020). Validation of the Spanish version of the Uterine Fibroid Symptom and Quality of Life (UFS-QoL) questionnaire in women with uterine myomatosis. Med Clin.

[19] Yeung SY, Kwok JWK, Law SM, Chung JPW, Chan SSC (2019). Uterine Fibroid Symptom and Health-related Quality of Life Questionnaire: a Chinese translation and validation study. Hong Kong Med J = Xianggang yi xue za zhi.

[20] Wang XQ, Zhu L, Xu T, Zhang L, Lyu T, Chen R (2017). Validation of the Chinese version of the uterine fibroid symptom and health-related quality of life. Zhonghua Fu Chan Ke Za Zhi.

[21] Coyne KS, Soliman AM, Margolis MK, Thompson CL, Chwalisz K (2017). Validation of the 4 week recall version of the Uterine Fibroid Symptom and Health-related Quality of Life (UFS-QOL) Questionnaire. Curr Med Res Opin.

[22] Fan S, Hu Z, Hong H, Zhao F (2012). Cross-cultural adaptation and validation of simplified Chinese version of the Roland-Morris Disability Questionnaire. Spine.

[23] Zhou X, Fang T (2020). A verification study on the reliability and validity of Chinese translation of uterine fibroid symptom and health-related quality of life. Nurs Pract Res.

[24] Chen J, Li Y, Wang Z, McCulloch P, Hu L, Chen W, Liu G, Li J, Lang J (2018). Evaluation of high-intensity focused ultrasound ablation for uterine fibroids: an IDEAL prospective exploration study. BJOG Int J Obstet Gynaecol.

[25] Li J, Wang H (2009). Evaluation on reliability and validity of SF-36 Scale (Version 2) in urban residents-quality of life in Chongqing. J Fourth Mil Med Univ.

[26] Li L, Wang HM, Shen Y (2003). Chinese SF-36 Health Survey: translation, cultural adaptation, validation, and normalisation. J Epidemiol Community Health.

[27] Chang CY, Huang CK, Chang YY, Tai CM, Lin JT, Wang JD (2010). Cross-validation of the Taiwan version of the Moorehead-Ardelt Quality of Life Questionnaire II with WHOQOL and SF-36. Obes Surg.

[28] Ware J, Snoww K, Ma K, Bg G (1993). SF36 Health Survey: manual and interpretation guide.

[29] Coyne KS, Margolis MK, Murphy J, Spies J (2012). Validation of the UFS-QOL-Hysterectomy Questionnaire: modifying an existing measure for comparative effectiveness research. Value Health.

[30] Coyne KS, Margolis MK, Bradley LD, Guido R, Maxwell GL, Spies JB (2012). Further validation of the uterine fibroid symptom and quality-of-life questionnaire. Value health.

[31] Revicki DA, Cook KF, Amtmann D, Harnam N, Chen WH, Keefe FJ (2014). Exploratory and confirmatory factor analysis of the PROMIS pain quality item bank. Qual Life Res.

[32] Patient-reported outcome measures: use in medical product development to support labeling claims. https://www.fda.gov/regulatory-information/search-fda-guidance-documents/patient-reported-outcome-measures-use-medical-product-development-support-labeling-claims 10.1186/1477-7525-4-79 PMC1629006 17034633

[33] Cohen J, Cohen J, Cohen JW, Cohen J, Cohen J, Cohen J, Cohen J, Cohen JW, Cohen N, Cohen C (1988). Statistical power analysis for the behavioral science. Technometrics.

[34] Ghanbari A, Hasandoost F, Lyili EK, Khomeiran RT, Momeni M (2017). Assessing emergency nurses' clinical competency: an exploratory factor analysis study. Iran J Nurs Midwifery Res.

[35] Pourmovahed Z, Mahmoodabadi HZ, Ardekani SMY, Fallahzadeh H, Tavangar H, Mahmoodabad SSM (2018). Validation of the Family Stability Questionnaire in married couples: a confirmatory factor analysis. Electron Phys.

[36] McHorney CA, Tarlov AR (1995). Individual-patient monitoring in clinical practice: are available health status surveys adequate?. Qual Life Res.

[37] Administration FaD: Guidance for Industry on Patient-Reported Outcome Measures: Use in Medical Product Development to Support Labeling Claims, vol. 65132–65133. Federal Register: 74; 2009. 10.1186/1477-7525-4-79 PMC1629006 17034633

[38] Pakpour AH, Zeidi IM, Saffari M, Burri A (2013). Psychometric properties of the Iranian version of the Sexual Quality of Life Scale among women. J Sex Med.

[39] Lyu J, Shen X, Hesketh T (2020). Sexual knowledge, attitudes and behaviours among undergraduate students in China—implications for sex education. Int J Environ Res Public Health.

[40] Sun X, Li C, Jin L, Fan Y, Wang D (2011). Development and validation of Chinese version of female sexual function index in a Chinese population—a pilot study. J Sex Med.

