# Supplementary material for: Adaptability and clinical applicability of UFS-QoL in Chinese women with uterine fibroid
Source: BMC Womens Health. 2022 Sep 10;22:372. doi: 10.1186/s12905-022-01963-z (PMC9463796; doi:10.1186/s12905-022-01963-z)
Supplement: Supplementary file 1 — Additional file 1. Table S1. Internal consistency of Self-consciousness stratifies by demographics (Cronbach’s alpha)*. [file 12905_2022_1963_MOESM1_ESM.docx]

**Supplement material**

**Table S1**. Internal consistency of Self-consciousness stratifies by demographics (Cronbach’s alpha) *

|  |  | Baseline | | 6-month | | 12-month | |
| --- | --- | --- | --- | --- | --- | --- | --- |
|  |  | n | α | n | α | n | α |
| Age | |  |  |  |  |  |  |
|  | <45 (n=1575) | 1575 | 0.56 | 1403 | 0.60 | 1345 | 0.61 |
|  | ≥45 (n=836) | 836 | 0.57 | 485 | 0.56 | 473 | 0.54 |
| Highest Level of Education | |  |  |  |  |  |  |
|  | Below junior | 352 | 0.56 | 246 | 0.64 | 232 | 0.61 |
|  | Junior school and above | 2057 | 0.58 | 1642 | 0.58 | 1586 | 0.59 |
| Family Annual Income, CNY | |  |  |  |  |  |  |
|  | < 50,000 | 613 | 0.54 | 457 | 0.53 | 441 | 0.61 |
|  | ≥ 50,000 | 1768 | 0.57 | 1418 | 0.60 | 1364 | 0.58 |
| Number of pregnancies | |  |  |  |  |  |  |
|  | 1 pregnancy | 1520 | 0.57 | 1216 | 0.59 | 1169 | 0.57 |
|  | >1 pregnancy | 891 | 0.55 | 672 | 0.57 | 649 | 0.62 |

^*^ >0.70 = satisfactory internal consistency

Abbreviation: CNY= China Yuan

**Table S2** Comparison of each item in subscale distribution in Factor analysis

| Subscales | Parents questionnaires validation | Chinese original validation | Validation |
| --- | --- | --- | --- |
| Symptom severity | 1 to 8 | 1 to 8 | 1 to 8 |
| Concern | 9, 15, 22, 28, 32 | 15、22、28、29、32 | 15, 22, 28, 29, 32, |
| Activities | 10, 11, 13, 19, 20, 27, 29 | 9、10、11、13、19、20、27 | 9 10 11, 12, 13 14 16 20, |
| Energy/Mood | 12, 17, 23, 24, 25, 31, 35 | 12、17、23、24、25、31、35 | 17, 19, 23, 24, 25,26, 27, 30, 31, 34, 35, |
| Control | 14, 16, 26, 30, 34 | 14、16、26、30、34 | 33 |
| Self-consciousness | 18, 21, 33 | 18、21、33 | 18, 21 |
| Sexual function | 36, 37 | 36、37 | 36, 37 |

| χ^2^/DF^*^ | RMSEA | NFI | CFI | TLI |
| --- | --- | --- | --- | --- |
| 8.101 | 0.077 | 0.942 | 0.842 | 0.894 |

**Table S3**. Construct validity of the UFS-QOL in confirmatory factor analysis

Abbreviation: UFS-QOL = Uterine Fibroid Symptom and Health-related Quality of Life; RMSEA=Root mean square error of approximation; NFI= normed fit index; CFI= comparative fit index; TLI=Tucker Lewis index

|  | Floor effects (0) | Ceiling effects (100) |
| --- | --- | --- |
| Symptom severity | 170 (7.05) | 0 (0) |
| Concern | 6 (0.25) | 210 (8.71) |
| Activities | 1 (0.04) | 228 (9.46) |
| Energy/mood | 3 (0.12) | 165 (6.84) |
| Control | 1 (0.04) | 124 (5.14) |
| Self-consciousness | 7 (0.29) | 366 (15.18) |
| Sexual functioning | 12 (0.5) | 409 (16.96) |
| Total HRQL score | 0 (0) | 29 (1.2) |

**Table S4**. Ceiling and Floor effects of UFS-QOL in baseline.
